# Advances in Liposome-Encapsulated Phthalocyanines for Photodynamic Therapy

**DOI:** 10.3390/life13020305

**Published:** 2023-01-21

**Authors:** Jakub Rak, Martina Kabesova, Jiri Benes, Pavla Pouckova, David Vetvicka

**Affiliations:** 1 LabInstruments s.r.o., Kralovicka 1430/1, 323 00 Pilsen, Czech Republic; 2 Institute of Biophysics and Informatics, First Faculty of Medicine, Charles University, Salmovska 1, 120 00 Prague, Czech Republic

**Keywords:** cancer, liposome, PDT, photosensitizer, phthalocyanine

## Abstract

This updated review aims to describe the current status in the development of liposome-based systems for the targeted delivery of phthalocyanines for photodynamic therapy (PDT). Although a number of other drug delivery systems (DDS) can be found in the literature and have been studied for phthalocyanines or similar photosensitizers (PSs), liposomes are by far the closest to clinical practice. PDT itself finds application not only in the selective destruction of tumour tissues or the treatment of microbial infections, but above all in aesthetic medicine. From the point of view of administration, some PSs can advantageously be delivered through the skin, but for phthalocyanines, systemic administration is more suitable. However, systemic administration places higher demands on advanced DDS, active tissue targeting and reduction of side effects. This review focuses on the already described liposomal DDS for phthalocyanines, but also describes examples of DDS used for structurally related PSs, which can be assumed to be applicable to phthalocyanines as well.

## 1. Introduction

### Basic Mechanism of PDT

In the modern era, photodynamic therapy (PDT) was initially discovered as a bactericidal treatment, and soon after it was first tested as an anti-cancer therapy [1] (preceding the development of radiotherapy); later, PDT found its clinical use in other areas, such as dermatology and ophthalmology. As a modality for the treatment of superficial tumours, it provides improved selectivity against diseased tissues compared to other cancer treatments (surgery, chemotherapy or radiotherapy). The technique uses photosensitizing agents (PSs) that can be light-activated. Activated PSs produce reactive oxygen species (ROS), which are able to destroy malignant cells. The primary selectivity for tumour cells is based on the greater accumulation of generally lipophilic PSs in malignant cells versus healthy ones, as well as the greater mortality of malignant versus healthy cells when exposed to oxidative stress [2,3,4]. 

**Figure 1:**
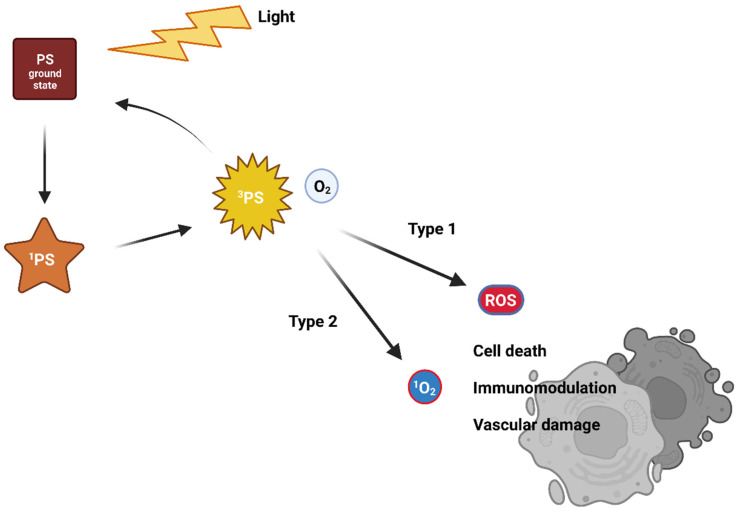
The traditional mechanism of action of photodynamic therapy.

Visible light of a suitable wavelength is used to activate the PS from its ground singlet state with no unpaired electron spins [5,6,7]. The excitation leads to a very unstable and short-lived singlet-excited state [7,8]. As it is very unstable, the PS, therefore, releases excess energy on the nanosecond time scale. For this, it uses photon emission (fluorescence) or internal energy conversion (heat) followed by returning again into the unexcited state. Another mechanism uses the transformation of an excited singlet state to an excited triplet state via one electron spin inversion [9]. The excited triplet state stability is higher compared to the excited singlet state, and so its lifetime is longer. The return to the ground state occurs via a photon emission (phosphorescence—rare at body temperature) or intersystem crossing or transfer of energy to the environment. Interactions with surrounding molecules follows two traditional reaction pathways, called Type I and Type II (Figure 1). With molecular oxygen, an excited PS generates reactive oxygen species (ROS). ROS likely react with surrounding organic molecules, causing oxidative damage that leads to cell death [10,11].

In the case of a Type I reaction, the excited-triplet-state PS reacts via an electron transfer with an electron donor, generating a radical anion of the PS. Radicals of PSs can react with dissolved oxygen and generate various ROS (superoxide anion O_2_^–^, peroxide anions, hydroxyl radicals OH^•^, or hydrogen peroxide H_2_O_2_). These then damage cellular compartments. However, oxygen is not absolutely necessary, as the free radicals of PSs can directly cause damage to biomolecules and cell compartments via radical chain reactions [12,13,14,15].

The Type II reaction is called triplet-triplet annihilation. The excited-triplet-state PS can react readily with molecules in triplet ground state. One such molecule is molecular oxygen. The excited PS transfers its energy directly to oxygen to yield excited singlet oxygen [16]. It is highly reactive and cytotoxic, causing irreversible cell damage [7,8].

**Figure 2:**
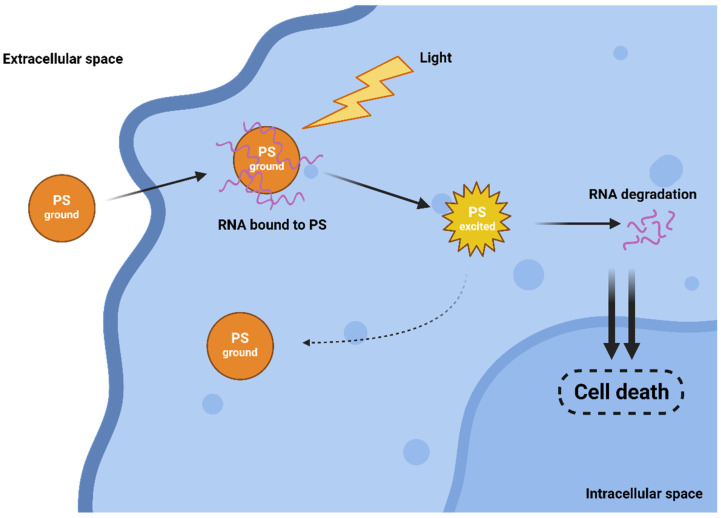
Oxygen-independent Type III mechanism of action.

The efficiency of both traditional Type I and II mechanisms is driven by the presence of oxygen, which can be a limiting factor as tumours are often hypoxic. Interestingly, in 2010, a novel and completely oxygen-independent mechanism was described [17]. In this mechanism, excited PSs directly degrade key cellular components, such as proteins and nucleic acids. For example, Yao and colleagues reported the synthesis of novel PSs NBEX, based on Nile Blue, which selectively bind to intracellular RNA, which is then destroyed upon PS excitation (Figure 2) [18]. For such oxygen-independent photoinactivation, the term Type III photochemical pathway is used [19].

Nevertheless, it is believed that the prevalent PDT mechanism is the Type II; however, the balance between all types of reactions depends on the nature of the PS [7,12,13,14,15,20,21,22]. When the ROS are generated, the PS either returns to the ground state, or the oxidation leads to photobleaching and destruction of the PS molecule [23]. If the PS returns to its ground state, it can be repeatedly activated by light [2,9,24,25,26].

## 2. Photosensitizers

The first use of PDT can be traced back to ancient Egypt more than 4000 years ago [27], but modern PDT appeared in the beginning of the twentieth century, when haematoporphyrin (Hp), the first PS, was isolated. This first generation of PSs showed weak absorption in the phototherapeutic window (range of wavelengths where light penetrates the tissues into the maximum depth) and prolonged the photosensitivity of the patient. This motivated research into better novel PSs designed to overcome the disadvantages of the first generation of PSs [28,29].

**Figure 3:**
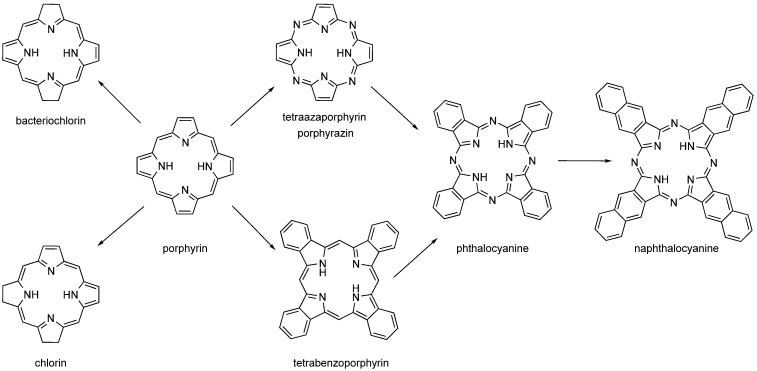
Structures of porphyrin-like compounds (chlorin, bacteriochlorin, porphyrin, tetraazaporphyrin, tetrabenzoporphyrin, phthalocyanine and naphthalocyanine).

The second-generation PSs are based on the structural motif of porphyrins, expanded porphyrins, and structures where aromatics are fused to pyrrole rings such as phthalocyanines (PCs), naphthalocyanines (NPCs), and benzoporphyrins (Figure 3). Many of these PSs are used in the form of metallocomplexes with diamagnetic ions. Porphyrins’ metabolic precursors, such as 5-aminolevulinic acid or its esters, are also used [2,4,30,31].

Photosensitivity and the ability to generate ROS are not rare, but still most of the PSs used in medicine are structural derivatives of cyclic tetrapyrroles. Cyclic tetrapyrroles exhibit low or no toxicity due to their natural similarity to endogenous structures occurring in the human body. At the same time, they show high yields in ROS production due to high absorption coefficients in the area of the phototherapeutic window, as well as fast blood clearance and specific tumour accumulation thanks to the EPR effect [4,32].

While benzoporphyrins are derived from porphyrin by fusion of benzene rings to each pyrrole unit, PCs are similarly derived from porphyrazine (pyrroles linked by nitrogen atoms instead of carbons in the porphyrin). NPCs are analogues of PCs with naphthalenes fused to pyrroles. However, PCs and NPCs are usually not used in their basic form, but as metallocomplexes with cations coordinated to the centre of the macrocycle. There are two reasons for this. The first, purely technical, synthesis takes place by successive addition of pyrrole units. While linear polymers are formed in the absence of a metal cation, in the presence of a metal cation these oligomers wrap around a central metal ion and readily form a macrocycle with an ion captured in the centre (or slightly above/below the macrocyclic plane). The coordinated central metal ion determines the photophysical properties of the PC and NPC. Diamagnetic transition metal ions (e.g., aluminium, zinc, and gallium) typically lead to complexes with high singlet oxygen quantum yields, while paramagnetic metal ions typically reduce lifetimes of excited states and lose their photoactivity [29,33,34,35,36,37,38].

In comparing PCs with NPCs, the great advantage of NPCs is stronger absorption in the near-infrared (NIR) region, due to which they have the potential to be used for highly pigmented tumours, where the penetration depth of visible light is reduced compared to common tumours. However, their clinical use is practically impossible due to their tendency to form photoinactive aggregates in solution, and their stability is not high enough (decomposition in the presence of light and oxygen). The advantages of PCs are chemical stability and resistance against (photo)chemical degradation [2].

In recent years, no PCs have been included in clinical trials [39], but several of their metallocomplexes have. Examples of such MPCs are the mixture of sulfonated aluminium derivatives of PCs called Photosense, developed and clinically approved in Russia [39,40], the silicon complex PC known as Phthalocyanine 4 (studied for sarcomas, cutaneous T-cell non-Hodgkin lymphoma, actinic keratosis, Bowen’s disease, mycosis fungoides/Sezary syndrome,) [39,41,42,43], phthalocyanine dental mouthwash Phtalox (iron phthalocyanine chloride) studied for periodontal diseases (periodontitis, bone loss) and intensely studied for preventing infection and progression of COVID-19 [39,44]. One more study using novel phthalocyanine dye (not specified) for periodontitis and alveolar bone loss is under preparation [39]. 

## 3. Effect on Cellular Level

The high reactivity of ROS causes them to react with the nearest cellular components. Studies report a radius of action ranging from 20–200 nm from where ROS are generated, while cell diameters are between 10 to 100 μm [3,4,45,46]. High levels of oxidative damage lead to cell death via autophagy, apoptosis or necrosis [47,48,49,50]. The predominant mechanism of cell death is determined by the subcellular localization of ROS generation and subsequent cellular targets of ROS [3,51]. Among these subcellular targets are most often the endoplasmic reticulum, mitochondria, lysosomes and the plasma membrane [4,52,53,54]. Molecular targets of ROS are mainly the thiol groups of proteins, membrane lipids and DNA [4,55].

## 4. Effect on Tumour Level

Unlike cell death, the tissue damage mechanisms are complex processes depending on the properties of the PS used and conditions of treatment. Most PSs are lipophilic macromolecular drugs, and as such, should tend to selective accumulation in tumour tissues due to the EPR effect. As tumour cells are rapidly growing, the formation of new blood vessels is stimulated. Consequently, the rapid growing of blood vessels leads to abnormalities in their architecture made up of misaligned defective endothelial cells, leading to a leaky vasculature that, hand in hand with often limited lymphatic drainage of tumours, results in the accumulation of high molecular weight compounds. However, the low success of nanomedicines in clinical trials gave rise to recent voices questioning this over-35-year-old phenomenon [56]. Broad analysis of the drug delivery literature from years 2005 to 2015 revealed very low accumulation rates, with an overall median of only 0.7% of intravenously administered nanoparticles actually delivered into the tumours [57]. Further, Sindhwani and colleagues reported that the vast majority of tested nanoparticles entered tumours via active transcytosis by endothelial cells. Additionally, they showed that the frequency of gaps in neovascular walls is too low to support the EPR effect [58]. It is also true that some tumours have hindered the features of the EPR effect due to their physiology. For example, pancreatic ductal adenocarcinomas (PDACs) are known for their dense desmoplastic stroma, making up to 90% of the tumour mass. In addition, PDACs are often hypoxic with very low neoangiogenesis, making it very hard for drug delivery systems to do their job [59,60]. However, Maeda, one of the fathers of the EPR effect, discussed these controversies in his recent article [61], giving reasonable arguments for his theory.

**Figure 4:**
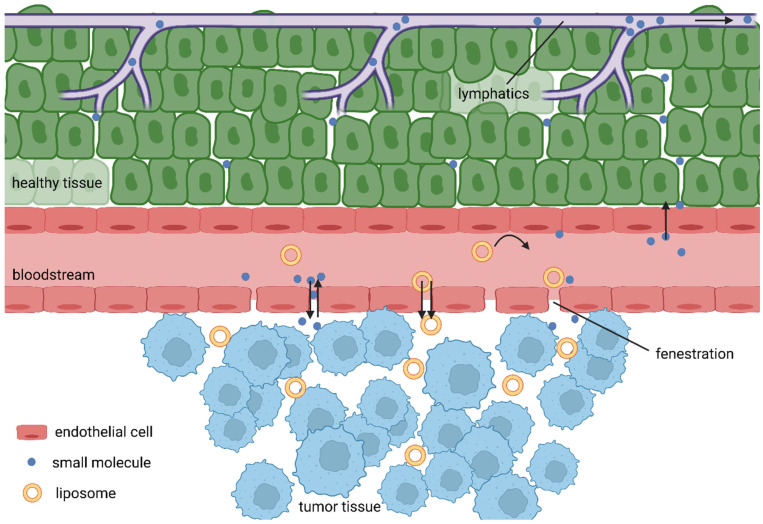
EPR effect.

Added to this are the differences in pH, specific expression of receptors and enzymes, then leading to the accumulation of PSs inside tumour cells (Figure 4). There is an additional mechanism that could increases the accumulation of PSs in close proximity to tumour cells, as common transport of lipophilic PSs in the bloodstream is often achieved by binding to lipoproteins and, at the same time, tumour cell membranes display a disproportionately high number of receptors for low-density lipoproteins [9,62,63,64]. PDT also has an impact on the vasculature, although the mechanism is not completely clear, and apparently a number of opposing processes might take place, the dominant among them including vascular leakage, stasis and collapsing blood vessels, causing ischaemic necrosis [4,65,66,67].

Besides the above mechanisms, yet another interesting effect that PDT has on the host antitumour immune reaction has been revealed in recent years. Many anticancer treatment regimes, including PDT, can awaken the immune system to help eradicate tumours by triggering immunogenic cell death (ICD). The activation of the immune system is preceded by signals from dying cells such as the translocation of intracellular damage-associated molecular patterns (DAMPs) such as heat shock proteins 70 and 90 (HSP70 and HSP90) and calreticulin (CRT) on the cell surface, or the release of high mobility group box 1 (HMGB1). The interaction of DAMPs and the particular receptors accelerates the phagocytosis of tumour antigens, leading to efficient cross-presentation to T cells [68,69].

Examples of PSs able to induce ICD include various phthalocyanines [70,71], Hypericin [72], Foscan [73], and Protoporphyrin IX [74], and the list keeps growing. Another example of a PS suitable for eliciting ICD is 8-methoxypsoralen (8-MOP). 8-MOP-UVA killed murine melanoma cells show signs of ICD, such as translocation of CRT on the plasma membrane and release of HMGB1, ATP, and type I interferon (IFN) [75]. Recent efforts sought to utilize this secondary immunotherapeutic feature of PDT in combination with primarily immunotherapeutic interventions in an attempted synergistic approach. Agostinis and colleagues successfully combined DC vaccines with a hypericin-based photodynamic to treat high-grade glioma (HGG) in animals [76]. Further, the combination of chemotherapy with PDT and CD73 blockade elicited strong and systemic antitumour immunity [77]. 

The general advantages of PDT compared to conventional cancer treatment (surgery, chemo- or radiotherapy) are mainly the absence of negative side effects, a low level of invasiveness and high tumour site specificity. The disadvantage is the limitation to superficial oncologic lesions with tumour thickness < 2–3 mm, because light in the range of visible wavelengths has only limited tissue penetration efficiency [78,79]. For a long time, PDT was, therefore, limited to use mainly for dermatology; however, coupling light sources with optical fibres allowed the treatment of deeper tumours such as those in the brain, colon, stomach, urinary bladder and other deeply located tumours [4]. In the case of tumour therapy, a single surviving cancerous cell means a failure of treatment. Like other conventional cancer approaches, PDT does not always achieve perfect results. However, it can be advantageously combined with other therapeutic techniques, such as surgery or chemotherapy. PDT has been successfully used to support surgical treatment by photosterilizing the tumour bed after surgical resection of a large neoplasm. Several studies have shown synergistic effects by combining PDT with low doses of chemotherapy drugs, which reduces side effects due to the lower doses of the chemotherapy drugs while killing cancer cells more effectively [4,80,81].

## 5. Drug Delivery Systems

PSs of the second generation are relatively non-toxic, and although they passively accumulate in tumour tissue thanks to the EPR effect, biodistribution still prevents a high proportion of administered PS from being used effectively and unnecessarily burdens the organism. Over the decades, a number of DDSs have been developed to increase the selectivity and improve the biodistribution of various drugs. A number of them were also used for PSs [14,15,30,82,83,84,85,86]. Considering the limitations of the drug approval process, the most promising DDSs are derived from liposomes.

## 6. Liposomes

Liposomes are the group of most frequently used DDSs for PDT and especially for PCs and MPCs due to their adaptive flexibility towards different physicochemical properties of various PSs and high loading. Liposomal formulations significantly improve the efficacy and safety of PSs, but conventional liposomes exhibit a short plasma half-life that limits effective tumour uptake. This can be improved by liposomes with a specifically modified design that circulate for a long time. In the same way, actively targeted liposomes can be prepared in order to improve their tumouritropic properties. These liposomes can preferentially extravasate from tumour blood vessels, exploiting the EPR effect [82].

Conventional liposomes are bilayered phospholipid vesicles separating the outer aqueous phase from the inner aqueous compartment. In addition to phospholipids, cholesterol is often added, which improves membrane stiffness, increases the stability of liposomes in the biological fluids, and reduces permeability for encapsulated molecules [83]. The size and properties can be easily modulated by the choice of phospholipids and the method of preparation (temperature, agitation, ultrasound, etc.). Popular phospholipids are, e.g., dipalmitoylphosphaditylglycerol (DPPG), dipalmitoylphosphatidylcholine (DPPC), DPPC/DPPG, DPPC-yolylcholesterol, 1,2-dioleoylphosphatidylserine (OOPS), and 1-palmitoyl-2-oleoylphosphatidylcholine (POPC) [84,85,86,87,88,89,90,91].

PSs are easily incorporated into the nascent membranes during preparation of liposomes. Hydrophobic PSs such as PCs and MPCs tend to aggregate in aqueous media. However, only monomers (and rarely dimers) can be activated in a manner leading to ROS production by the mechanisms described above. Aggregation thus dramatically reduces their efficiency. Incorporation of PSs into liposome membranes effectively suppresses aggregation and significantly increases the ability to produce ROS [92]. However, the stability of conventional liposomes in the bloodstream is limited due to the exchange of lipids between lipoproteins and liposomes. This forces irreversible liposome disintegration and release of PSs. The lipophilic PS released into the bloodstream then usually binds to lipoproteins and transport plasma proteins such as albumin. A typical plasma half-life is in the range of minutes [93]. Plasma proteins not only function as carriers of PSs and other lipophilic molecules, but also readily opsonize conventional liposomes. These are then taken up by mononuclear phagocyte cells and concentrate in phagocyte-rich tissues (bone marrow, liver, spleen) [94].

Lipoproteins in plasma not only cause the breakdown of liposomes, but also serve to transport PSs (e.g., Zn(II) phthalocyanine, Sn(IV) naphthalocyanine or a benzoporphyrin derivative), especially low-density lipoproteins (LDLs) [95,96,97,98]. This is important for targeting tumour cells. The rapid proliferation of cancerous cells increases their demands on cholesterol for membrane synthesis and, therefore, these cells express an increased number of LDL receptors [99]. Thus, endocytosis mediated by LDL receptors is one way to increase uptake of PSs by tumours.

## 7. Passively Targeted Liposomes for PDT

Rapid neoangiogenesis in cancerous tissue stimulates the formation of new blood vessels; however, these are defective with fenestrae of a pore size of 100–1200 nm, which leads to an enhanced vascular permeability [100]. Due to the lack of effective lymphatic drainage, extravasated macromolecules do not return to the central circulation efficiently. Together, they cause the EPR effect [101]. When they are circulating for a sufficiently long period, the EPR effect allows PSs to passively accumulate in tumour tissue. Therefore, liposomes should be designed as stealth for the reticuloendothelial system [102]. 

Incorporation of PCs and MPCs into liposomes is widely used; examples include hydroxyl-aluminium phthalocyanine [90,91], aluminium chloride phthalocyanine [103,104], zinc phthalocyanine [105,106] or phthalocyanine conjugates with gold nanoparticles [107]. However, blood clearance of conventional liposomes happens typically in tens of minutes. Suitable surface modifications can produce long-circulating liposomes with half-lives of hours or tens of hours. For example, glycolipids (e.g., monosialoganglioside (GM1)) or lipids with polyethylene glycol head groups significantly help to prolong half-lives beyond 10 hours [93,108]. They are sometimes called stealth liposomes [100,109].

## 8. Actively Targeted Liposomes 

Conventional liposomes provide only passive targeting. Therefore, liposome constructs that utilize specific targeting moieties have been developed. These moieties are bound to the liposomal outer layer, facilitating selective binding to targeted tissues or cells. Such moieties can be anything with high selective affinity to specific markers and sufficiently expressed on the membrane of tumour cells. Biological components capable of recognizing the tumour can be integrated with conventional liposomes, but the advantage is in integration with long-circulating liposomes [82,110,111,112].

## 9. Antibody-Modified Liposomes

The immunoliposome approach uses monoclonal antibodies (mAbs) and mAb fragments that are conjugated to liposomes. A typical liposome has the capacity to carry thousands of PS molecules, whereas individual antibodies in immunoconjugates can carry a much smaller number of PS molecules [82]. The advantage of mAbs over most other tumouritropes is a high degree of specificity, but they can cause immune reactions. The use of mAb fragments lacking the Fc part of the mAb maintains high specificity, while the immune reaction is minimized by preventing the phagocytosis [113].

Examples of the use of this approach for targeted drug delivery of PCs and MPCs include the use of mAb-liposomes containing tetrasulfonated aluminium phthalocyanine specifically targeting CFU-GM progenitor cells [114] or T-lymphocytes [114], a mixture of sulfonated aluminium phthalocyanines encapsulated in mAb-liposomes selective against human bladder cancer cells [115], or zinc phthalocyanine containing tumour-targeted liposomes [116].

There are two recent works that did not use mAb-targeted liposomes for PCs; however, it can be expected that this will be the next step. In the first work, they used NaYF_4_:Yb,Er upconversion nanoparticles covalently bound to zinc tetracarboxyphenoxy phthalocyanine and immunoconjugated with a specific mAb selective for HER2-overexpressing malignant cells [117]. In the second work, they prepared NaYF_4_:Yb,Er upconversion nanoparticles with the chemotherapeutic agent doxorubicin and PS methylene blue entrapped in nanoliposomes coated with anti-HER2 peptide [118]. Since NaYF_4_:Yb,Er upconversion nanoparticles are most often used with phthalocyanines [30,119,120], and HER2-targeted liposomes have been prepared, there is an opportunity to combine these two systems.

## 10. Ligands

A popular strategy for molecularly targeted drug delivery uses tumour-targeting peptides, proteins and glycoproteins. Although the direct conjugation of PCs and MPCs with tumour-targeting peptides has been described, and the incorporation of PCs and MPCs into liposomes is widely used, peptide-targeted liposome formulations for PCs or MPCs are rarely used. The first pioneer used transferrin-conjugated liposome targeting of aluminium phthalocyanine tetrasulfonate to rat bladder carcinoma cells [121,122]. Transferrin was used because the transferrin receptor is overexpressed in many tumour cells due to their increased iron requirement.

Vitamins such as folic acid can be used to target tumour cells, as numerous human carcinomas often overexpress the folate receptor on their plasma membrane. Binding of folate conjugated to the delivery system to its receptor on the cancer cell triggers active endocytosis [123]. Two decades ago, chloro-aluminium phthalocyanine tetrasulfonate was targeted using folate-conjugated liposomes [124]. Since then, more sophisticated systems have been developed. Recently, folate-conjugated liposomes have been used for targeted delivery of zinc phthalocyanine coupled with graphene quantum dots (using the fluorescence resonance energy transfer (FRET) as a mechanism to kill the cancer cells) with catalytic Pt nanoparticles decorated with MnO_2_ (converting H_2_O_2_ to O_2_) for tumour treatment in hypoxic conditions [125]. Hypoxia dramatically increases the level of ROS in tumour cells compared to healthy cells (up to 100 × 10^−6^ M and ≈20 × 10^−9^ M, respectively) [126]. A similar approach uses a combination of MnO_2_ nanoparticles (hypoxia), paclitaxel (chemotherapy) and chlorin e6 (PS) delivered in liposomes [127], or MnO_2_ nanoparticles with acriflavine and chlorin e6 encapsulated in pH-sensitive liposomes [128]. Another folate-modified liposomal nanophotosensitizer based on a zinc phthalocyanine showed high efficacy in vivo [129].

There are several other ligands that have proven suitable for targeting tumour cells that overexpress receptors for these ligands; however, they have not yet been used for targeting liposomes as carriers of PCs and MPCs. However, since they have already proven their effectiveness for liposomes carrying other PSs or as ligands directly conjugated to PSs, it can be expected that their use for PCs and MPCs in liposomes will also work. Examples are growth factors (epithelial or nerve growth factor) [130,131], lipoproteins [99], glycolipids and glycosylated cholesteryl derivatives [132]. Recently, hyaluronic acid-coated pH-sensitive liposomes for combined chemotherapy with mitochondria-targeted PDT have been prepared [133]. Similarly, the ligands mentioned in the previous paragraphs were also used for liposomes carrying PSs other than PCs, such as folate-targeted PEGylated liposomes [134] and micelles [135]. 

All mentioned ligands pose a minimal risk of inducing immune responses. However, compared to antibodies, the specificity towards tumour cells may be lower, as healthy cells often share the targeted structures [113].

## 11. Liposomes with Activable Release Mechanisms

Targeting of liposomes can be either passive or active, but in both cases, it is necessary to release the PS from the liposome before light irradiation. If it is enclosed in a liposome and exposed to light, an excited PS might oxidatively break down its carrier and auto-release itself. However, the PS wastes its oxidation capacity. As already described above, the PS activation and deactivation process is carried out by several mechanisms, including those that degrade the PS for further use (photobleaching). Depending on the probability of individual mechanisms determined by intrinsic properties of the PS and its environment, the PS can endure only a limited number of activation cycles before its destruction occurs. Moreover, due to the limited time of irradiation during treatment, the PS after auto-release may not have enough time to interact with cells or to get sufficiently close to the particular intracellular target.

**Figure 5:**
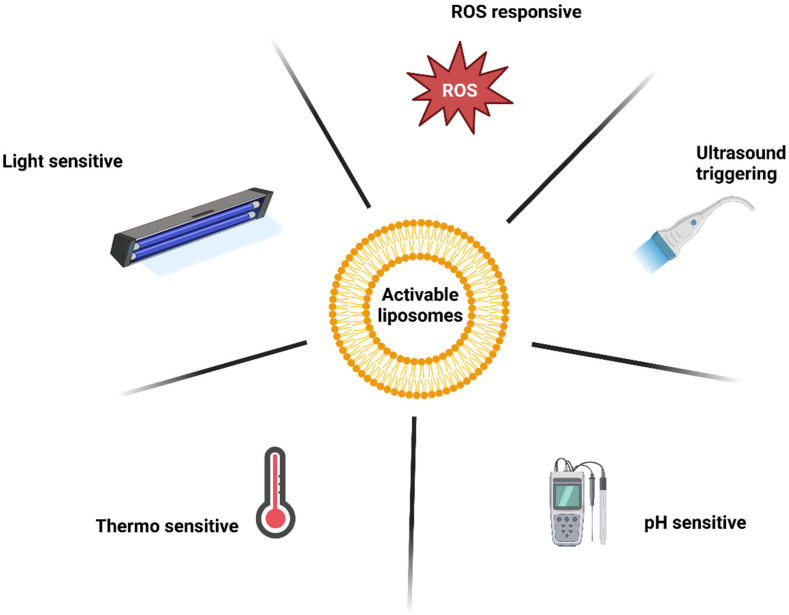
Mechanisms of liposome release activation.

For the reasons mentioned above, liposomes with an activable release strategy are advantageous. These liposomes release PSs upon a particular stimulus, so the PSs can interact with their surroundings. A combination with active tumour cell targeting may provide additional benefit. The various mechanisms for triggered release will be discussed further (Figure 5) [113].

## 12. Ultrasound

A limitation of PDT is the efficiency of tissue penetration by light within the visible wavelengths, so classical PDT is well-suited to thin and superficial tumours only. It is less known that many PSs are sensitive to ultrasound waves. These ultrasound-stimulated PSs can cause necrosis and cell death in a manner similar to light irradiation. This process is known as sonodynamic therapy (SDT) [136]. A combination of these two treatments is called sonophotodynamic therapy (SPDT). In recent study, zinc phthalocyanine in liposomes has been used for PDT, SDT, and SPDT. Combined SPDT is more effective than individual treatments and can reduce the required dose of PS, ultrasound and light. This reduces the side effects of the treatment. The most effective treatment arrangement is PDT, followed by SDT [137].

## 13. Fusogenic Liposomes

When liposomes target non-internalizing receptors, the contents of the liposome can be transferred into the cell when the liposome fuses with the cell’s plasma membrane. Liposomes with fusogenic viral proteins attached to their surface (virosomes) can be used for this purpose. An example is the protein coating of the Sendai virus, taking advantage of the ability of this virus to merge with nearly any mammalian cell. The main disadvantage of this approach is poor selectivity for tumour cells. To increase selectivity, fusogenic proteins must be shielded to a certain level; the addition of a targeting moiety is advantageous [138,139].

Distribution of therapeutics within the tumour is heterogenous, dictated by individual tumour microenvironment, often restricted to areas near the neovasculature. Extracellular vesicles (EVs) shedded by tumour cells are able to transport anticancer agents between individual cells, enabling, e.g., delivery into deeper layers. However, the natural generation of EVs is not efficient enough to use this approach. Recently, fusogenic liposomes containing monensin as a stimulant of EV secretion and PSs were described. First the fusogenic liposomes transfer monensin and PSs to the tumour cellular membrane. Through the shedding of EVs, PSs and monensin are secreted by the outer tumour cells, and both drugs are transferred deeper and deeper into the tumour mass [140]. A similar approach to understanding the mechanism was used here [141].

## 14. pH-Sensitive Liposomes

The pH-sensitive liposomes are so designed that their phospholipid bilayer is destabilized at a certain pH, most often within the pH range 5–6.3, because the pH in endosomes, lysosomes and also in tumour interstitium is reduced compared to physiological pH [142]. To achieve pH-induced destabilization, membranes must contain structures with charge around the neutral pH but that discharge when the pH turns acidic. In addition, pH-sensitive liposomes typically contain phosphatidylethanolamine as an auxiliary lipid [143].

Examples can be liposomes composed of phosphatidylethanolamine and cholesteryl hemisuccinate. Cholesteryl hemisuccinate is negatively charged at pH 7.4 and imparts a negative charge to the liposome. Upon acidification, its carboxyl group is protonated, and the repulsion between the two layers decreases, which in turn leads to a collapse of the lamellar structure [144]. Other examples include liposomes containing pH-sensitive diplasmenylcholine for the transport of aluminium phthalocyanine tetrasulfonate into lysosomes [124].

Recently, liposomes sensitive to pH were designed for the simultaneous delivery of porphyrin derivative (targeting mitochondria) and selected chemotherapeutics. Liposomes were additionally modified by hyaluronic acid to specifically target CD44, which is commonly overexpressed in colon cancer and enhances cellular uptake via receptor-mediated endocytosis [133].

A completely opposite approach is to use one wave of carriers to adjust the pH in the target and then a second wave to deliver the PSs that need the adjusted pH to be effective. To modulate the tumour microenvironment by selectively degrading the condensed extracellular matrix, collagenase-encapsulated nanoscale coordination polymers were synthesized. After intravenous application, the described nanocarriers show effective delivery to the tumour, where collagenase is released as the nanoscale coordination polymer structures collapse in the acidic tumour microenvironment. The released collagenase starts degrading collagens, loosening the tumour stroma, enhancing the tumour perfusion, and relieving the hypoxia. As a consequence, the next application of liposomes loaded with chlorin e6 shows improved accumulation in the tumour tissue [145]. Another complicated system is made up of doxorubicin and zinc phthalocyanine co-loaded mesoporous silica with calcium phosphate in PEGylated liposomes. Mesoporous silica nanoparticle pores are loaded with doxorubicin by diffusion; the cores are subsequently covered by calcium phosphate and encapsulated by liposomes loaded with zinc phthalocyanine, thus forming the final PEGylated liposomes. The calcium phosphate interlayer can be used to acquire controllable pH-sensitive release of doxorubicin. At physiological pH, calcium phosphate retains its mineral structure, while it dissolves at a lower pH in the lysosomes, so it serves as gatekeeper to achieve controllable pH-sensitive release of the payload and to circumvent premature release of doxorubicin and consequent undesired side effects. Besides, the chemotherapy using doxorubicin and PDT effect of phthalocyanine, calcium phosphate induces apoptotic cell death caused by increasing osmotic pressure with the endo/lysosomes thus improving the anticancer efficiency [146].

## 15. Light-Sensitive Liposomes

Chromophores naturally present in the body (e.g., haemoglobin) cause UV and visible light to penetrate only a few millimetres deep. In contrast, NIR penetrates to a depth of units to tens of centimetres and also does not damage the tissue like UV. On the other hand, lower NIR energy may not be sufficient to induce a physicochemical change leading to targeted drug release. Nevertheless, few systems of light-induced destabilization of liposome membrane have been developed. These usually use organic NIR-sensitive chromophores. Another popular option is inorganic upconverting nanoparticles (mixed lanthanide fluorides), which can convert two NIR photons into one photon of visible light. The wavelengths of both absorbed and emitted light can be modulated by the composition of the upconverting nanoparticle. The disadvantage of these nanoparticles is the fact that two NIR photons must be absorbed in a very short period of time, which requires high-intensity radiation sources (lasers). With regard to the maximum power of the lasers (so as not to damage the surface tissue) and the scattering of light during tissue penetration, the usable depth is effectively limited [147,148,149].

Photoisomerization utilizes the incorporation of a functional group that changes conformation upon illumination with light into the lipid bilayer. This change then leads to membrane destabilization. However, this isomerization usually requires high-energy radiation (UV, blue light), which limits its use in clinical practice due to the limited penetration of this radiation into the tissue. Popular moieties are based on azobenzene [150,151,152,153,154] or spiropyran [155].

Photocleavage uses the incorporation of an amphiphilic molecule into the liposomal membrane, which splits into polar and non-polar parts after illumination. The amphiphilic character is lost, and membrane destabilized. Although a number of synthetic amphiphiles suitable for this purpose have been prepared, cleavage of natural plasmalogens after photodynamic sensitization can be used with advantage. Zinc phthalocyanine after illumination with > 640 nm was used for the induced cleavage of plasmalogen [156,157]. Even higher wavelengths (800 nm) were used for splitting plasmalogen using tin octabutoxyphthalocyanine or bacteriochlorophyll a [157]. In general, wavelengths of light that allow deeper penetration of the skin and are generally more biologically friendly can be used during photolysis.

Photocrosslinking uses the polymerization of unsaturated bonds after irradiation. These unsaturated bonds are usually found in the hydrophobic part of the membrane. Irradiation and subsequent polymerization cause changes in morphology of membrane (local shrinkage). Similarly to photoisomerization, photocrosslinking requires rather higher energy radiation [147,148].

Another mechanism is light-induced oxidation. PSs entrapped in micelles after light activation and subsequent ROS production are likely to cause oxidative degradation of the lipid bilayer and PS release. However, ligands that undergo changes in response to light can also be intentionally added to the membrane. As a result of irradiation, photoalteration in the lipid membrane leads to increased permeability of the liposome for the incorporated photosensitizer [158].

Plasmenylcholine liposomes with membrane-incorporated PSs are an example. They use PSs to produce singlet oxygen, which is used for the oxidation of the plasmalogen vinyl-ether bond and subsequent production of single-chain surfactants derived from cleaved plasmenylcholine. ROS attack the plasmenylcholine-vinyl-ether bond and thus destabilize the liposome membrane and mediate liposome permeability [157]. A similar example of PS-induced destabilization of liposomes uses photooxidizable lipids in combination with haematoporphyrin ether as PS [159]. Very popular are porphyrin-phospholipid (PoP) liposomes containing porphyrin covalently bonded to a phospholipid side chain. These are commonly activated by 665 nm lasers to produce ROS that subsequently oxidize other lipids (such as dioleoylphosphatidylcholine (DOPC) and cholesterol) and lead to leakage of liposomes [160,161,162].

Recently, amphiphilic tetraethylene glycol-substituted zinc phthalocyanine has been incorporated into liposomal bilayer made of DPPC (1,2-dipalmitoyl-sn-glycero-3-phosphocholine). After light irradiation, the micelles become leaky and release doxorubicin encapsulated in the inner phase of a liposome [163]. 

Similarly, a light-sensitive liposome was prepared for the triggered release of doxorubicin, which was made up of a special phospholipid (1-(1z-octadecenyl)-2-oleoyl-sn-glycero-3-phosphocholine) and Indocyanine green (hydrophobically modified) as PS. The anti-Her2 antibodies were used to modify the liposomal outer layer to target Her2-overexpressing tumour cells [164].

Although light-induced oxidation and subsequent pore formation leads to increased membrane permeabilization, high selectivity of the pore to the given drug also plays a role in successful drug delivery. This was shown, for example, using trisulfonated aluminium phthalocyanine and zinc phthalocyanine tetrasubstituted with a glycerol moiety as sources of ROS and a set of fluorescent markers as drug models [165]. 

## 16. Thermo-Sensitive Liposomes

Thermally sensitive liposomes, when heated above the phase transition temperature of the liposome membrane, will dramatically increase membrane lipid disorder. This will subsequently increase the permeability of the liposomal bilayer and the release of PSs. As the tumour tissue needs to be heated, the liposomes’ phase transition temperature needs to be right above physiological body temperature. Suitable lipids for the construction of such liposomes are, for example, dipalmitoyl phosphatidylcholine and dipalmitoyl phosphatidyl glycerol. 

While classic thermal cancer therapy uses the higher sensitivity of tumour cells compared to healthy ones to elevated temperatures (42–45 °C), and thus, tissue must be locally heated, thermosensitive liposomes can be modulated to be sensitive to temperatures over 37 °C, so there is no need for the tissue to be heated so much. Conversely, in the case of simultaneous heating and illumination of the tumour by light, the combined effect of mild hyperthermia and rapid release of PSs from thermosensitive liposomes can be used [166].

Recently, NIR-activated thermosensitive liposomes with encapsulated cyanine dye as PS with a phase transition temperature of 42.3 °C were studied [167]. This work was a continuation of the previous work where thermosensitive liposomes self-assembled from DPPC, distearoylphosphatidylcholine (DSPC) and 3-sn–phosphatidylethanolamine-poly(ethylene glycol)2000 (DSPE-PEG2000) without loaded PS and displaying a phase transition temperature of 41.1 °C were prepared [168].

Another approach is the formulation of a eutectic mixture of lauric and stearic acids in a 4:1 ratio. Compared to the melting points the of parental components (44 °C and 69 °C), the eutectic mixture exhibits a sharp melting point at 39 °C. Composite structures based on this eutectic mixture with the NIR-absorbing dye indocyanine green as PS, doxorubicin, and liposomal shells labelled with folate and conjugated gadolinium chelate with enhanced magnetic resonance performance and active targeting were prepared. These composite structures have been designed for triple-modal imaging (magnetic resonance imaging, photoacoustic, fluorescence) combined with multimodal tumour therapy (PDT, chemo-, and photothermotherapy) [169].

## 17. ROS-Responsive Liposomes

ROS-responsive DDSs can potentially improve the therapeutic efficiency and decrease the adverse effects of antitumour therapies including combinations of modes of action (such as chemotherapy with PDT). 

A few ROS-responsive polymers derived from poly(propylene sulfide) [170], polythioether ketal [171], or copolymers containing selenium [172] were designed for drug delivery [173,174,175]. These polymers do not seem to be suitable for liposome formulations with clinical applications. 

An example of a liposome-based formulation could be the recently published indocyanine green with ROS-responsive doxorubicin prodrug encapsulated in liposomes coated with polyethylene glycol. Indocyanine green serves as both ROS trigger and PS. The produced ROS break oxidation-labile bonds of ROS-responsive prodrugs and disrupt liposomes, leading to leakage of the drugs at the same time. The leaked PS continues to produce ROS (PDT) [173].

## 18. Conclusions

This review builds on our earlier publication (Drug delivery systems for phthalocyanines for photodynamic therapy) and expands it with the latest publications. There are various promising types of drug delivery systems (DDS) for phthalocyanines, at least from an academic point of view. However, considering the drug approval process, liposomes are the closest to clinical practice. Although liposomes may seem to be an already well-researched tool for DDS, recent works show their possibilities in targeting and releasing content controlled by external stimuli (triggers such as ultrasound, temperature, pH, light, ROS). Nevertheless, this review is not limited only to existing combinations of liposomes with phthalocyanines, but also presents systems whose principles are also applicable to phthalocyanine-liposome combinations or their combination with other modes of action (e.g., chemo- or radiotherapy). Although representatives of phthalocyanines have already been approved for clinical use or are the subject of clinical studies, for the time being, they are simple drugs without the use of advanced DDSs. The use of DDSs such as liposomes with active targeting and triggered release mechanisms are still awaiting clinical application.

## Data Availability

Not applicable.
